# Finished Genome Assembly of Warm Spring Isolate *Francisella novicida* DPG 3A-IS

**DOI:** 10.1128/genomeA.01046-15

**Published:** 2015-09-17

**Authors:** Shannon L. Johnson, Timothy D. Minogue, Hajnalka E. Daligault, Mark J. Wolcott, Hazuki Teshima, Susan R. Coyne, Karen W. Davenport, James G. Jaissle, Patrick S. Chain

**Affiliations:** a Los Alamos National Laboratory (LANL), Los Alamos, New Mexico, USA; b U.S. Army Medical Research Institute for Infectious Diseases (USAMRIID), Frederick, Maryland, USA

## Abstract

We sequenced the complete genome of *Francisella novicida* DPG 3A-IS to closed and finished status. This is a warm spring isolate recovered from Hobo Warm Spring (Utah, USA). The final assembly is available in NCBI under accession number CP012037.

## GENOME ANNOUNCEMENT

*Francisella tularensis* subsp. *novicida* DPG 3A-IS was isolated in late December 2006 from Hobo Warm Spring, just north of Salt Lake City, Utah, USA (1). This isolate was recovered from the same area that strains U122 and UT01-4992 were isolated, in an attempt to further investigate *F. novicida* persistence and diversity (2, 3). By whole-genome mapping, the recent isolates of *F. novicida* from the hot springs are significantly different from the other *F. novicida* previously published, suggesting a greater species diversity than previously recognized. The sequences of the recent hot spring isolates will aid in the understanding of the status of *F. novicida* as a separate species from *F. tularensis* and the differences in virulence between *F. tularensis* and *F. novicida* in current animal models of tularemia disease.

High-quality genomic DNA of *F. novicida* DPG 3A-IS was extracted from a purified isolate using QIAgen Genome Tip-500. Specifically, 100-mL bacterial cultures were grown to stationary phase and nucleic acid was extracted per the manufacturer’s recommendations with one minor variation. Genomic draft data, including both short- (300 ± 70 bp) and long-insert (10,434 ± 1,922 bp) paired-end Illumina data, were trimmed for quality and reduced to a total genome coverage of 342× for the assemblies. All raw data have been deposited to NCBI and are available in the SRA (SRP060258) (4). Draft data were assembled using Newbler version 2.6, Velvet version 1/2/08, AllPaths version 44837, and parallel Phrap version SPS 4.24 to generate a single closed contig of finished quality (5–8).

The final assembly consists of one closed 1,898,140-bp circular contig with 32.3% G+C. Preliminary review of the annotations found 2,037 coding regions, 10 rRNA sequences, and 38 tRNA sequences.

### Nucleotide sequence accession number.

The full genome sequence for *Francisella novicida* DPG 3A-IS has been deposited in NCBI under accession number CP012037.

## References

[1] Whitehouse CA, Kesterson KE, Duncan DD, Eshoo MW, Wolcott M 2012 Identification and characterization of *Francisella* species from natural warm springs in Utah, USA. Lett Appl Microbiol 54:313–324. doi:10.1111/j.1472-765X.2012.03214.x. 22283482

[2] Brett ME, Respicio-Kingry LB, Yendell S, Ratard R, Hand J, Balsamo G, Scott-Waldron C, O’Neal C, Kidwell D, Yockey B, Singh P, Carpenter J, Hill V, Petersen JM, Mead P 2014 Outbreak of *Francisella novicida* bacteremia among Inmates at a Louisiana Correctional Facility. Clin Infect Dis 59:826–833. doi:10.1093/cid/ciu430. 24944231

[3] Molins-Schneekloth CR, Belisle JT, Petersen JM 2008 Genomic markers for differentiation of *Francisella tularensis* subsp. *tularensis* A.I and A.II strains. Appl Environ Microbiol 74:336–341. doi:10.1128/AEM.01522-07. 18024683 PMC2223193

[4] Bennett S 2004 Solexa Ltd. Pharmacogenomics 5:433–438. doi:10.1517/14622416.5.4.433. 15165179

[5] Butler J, MacCallum I, Kleber M, Shlyakhter IA, Belmonte MK, Lander ES, Nusbaum C, Jaffe DB 2008 ALLPATHS: *de novo* assembly of whole-genome shotgun microreads. Genome Res 18:810–820. doi:10.1101/gr.7337908. 18340039 PMC2336810

[6] Ewing B, Green P 1998 Base-calling of automated sequencer traces using Phred, II: error probabilities. Genome Res 8:186–194. doi:10.1101/gr.8.3.186. 9521922

[7] Ewing B, Hillier L, Wendl MC, Green P 1998 Base-calling of automated sequencer traces Using Phred, I: accuracy assessment. Genome Res 8:175–185. doi:10.1101/gr.8.3.175. 9521921

[8] Zerbino DR, Birney E 2008 Velvet: algorithms for de novo short read assembly using de Bruijn graphs. Genome Res 18:821–829. doi:10.1101/gr.074492.107. 18349386 PMC2336801

